# Leukocytes Classification for Leukemia Detection Using Quantum Inspired Deep Feature Selection

**DOI:** 10.3390/cancers15092507

**Published:** 2023-04-27

**Authors:** Riaz Ahmad, Muhammad Awais, Nabeela Kausar, Usman Tariq, Jae-Hyuk Cha, Jamel Balili

**Affiliations:** 1 Department of Computer Science, Iqra University, Islamabad 44800, Pakistan; 2 Department of Computer Science, COMSATS University Islamabad, Wah Campus, Wah 47010, Pakistan; 3 Department of Electrical & Computer Engineering, COMSATS University Islamabad, Wah Campus, Wah 47010, Pakistan; 4 Department of Management Information Systems, College of Business Administration, Prince Sattam Bin Abdulaziz University, Al-Kharj 16278, Saudi Arabia; 5 Department of computer Science, Hanyang University, Seoul 04763, Republic of Korea; 6 College of Computer Science, King Khalid University, Abha 61413, Saudi Arabia

**Keywords:** deep learning, feature selection, evolutionary algorithms, quantum-inspired, white blood cell classification, convolutional neural network (CNN)

## Abstract

**Simple Summary:**

In this work, an improved pipeline for Leukocytes subtype classification is proposed which uses transfer learning for deep feature extraction and a quantum inspired evolutionary algorithm for feature selection. The proposed system achieves a high accuracy with smaller number of features as compared to the classical methods.

**Abstract:**

Leukocytes, also referred to as white blood cells (WBCs), are a crucial component of the human immune system. Abnormal proliferation of leukocytes in the bone marrow leads to leukemia, a fatal blood cancer. Classification of various subtypes of WBCs is an important step in the diagnosis of leukemia. The method of automated classification of WBCs using deep convolutional neural networks is promising to achieve a significant level of accuracy, but suffers from high computational costs due to very large feature sets. Dimensionality reduction through intelligent feature selection is essential to improve the model performance with reduced computational complexity. This work proposed an improved pipeline for subtype classification of WBCs that relies on transfer learning for feature extraction using deep neural networks, followed by a wrapper feature selection approach based on a customized quantum-inspired evolutionary algorithm (QIEA). This algorithm, inspired by the principles of quantum physics, outperforms classical evolutionary algorithms in the exploration of search space. The reduced feature vector obtained from QIEA was then classified with multiple baseline classifiers. In order to validate the proposed methodology, a public dataset of 5000 images of five subtypes of WBCs was used. The proposed system achieves a classification accuracy of about 99% with a reduction of 90% in the size of the feature vector. The proposed feature selection method also shows a better convergence performance as compared to the classical genetic algorithm and a comparable performance to several existing works.

## 1. Introduction

White blood cells (WBCs), or leukocytes, are an essential part of the human immune system. They play a critical role in defending the body against infectious diseases and foreign substances by identifying and neutralizing pathogens, such as bacteria and viruses [1,2]. Leukocytes are of two types, i.e., agranulocytes and granulocytes. Granulocytes are so named because they contain granules in their cytoplasm. They are produced in the bone marrow and are involved in the immune response to various types of infections. There are three main types of granulocytes: neutrophils, eosinophils, and basophils. Agranulocytes, which are cytoplasmically granule-free cells, are further divided into two types, i.e., monocytes and lymphocytes [3]. Each type of cell plays a distinct role in the body’s immune system. For instance, neutrophils and lymphocytes combat viruses, bacteria, fungi, and other cells that endanger the body’s ability to function. Eosinophils play a role in the body’s normal immunological and inflammatory reactions. Monocytes fight against infections, kill the cancerous cells, and remove the dead or damaged tissues [4]. WBC analysis, classification, and counting are critical steps in the diagnosis of many diseases, particularly leukemia, a type of blood cancer caused by abnormal growth of malignant WBCs in the bone marrow. The conventional method for leukemia diagnosis is based on a series of blood tests, the majority of which involve visual examination of blood images by hematologists. Automated blood analysis has drawn a lot of research attention recently thanks to advancements in machine learning. Nevertheless, morphological overlap between several subclasses and their structural abnormalities makes machine learning-based categorization and localization of WBCs challenging. Among the modern approaches, deep learning with convolutional neural networks has shown significant promise [5,6]. Although deep neural networks have the ability to obtain a rich collection of features from images, their training requires a very large dataset, which is typically not available in medical imaging. In such a case, transfer learning is the preferred approach in which a pre-trained CNN is fine tuned for a specific task [7]. A number of well-performing CNNs have been made publicly available by the research community, including VGGNet [8], Resnet [9], Darknet [10], Mobilenet [11], Inception [12], and Xception [13] etc. Deep models have the potential to extract a wide variety of information from the images because of their aptitude for self-learning. As a result, a high level of accuracy is achieved for a range of image classification scenarios. In many applications based on deep transfer learning, feature selection is a crucial step, as it helps to reduce the dimensionality of the feature space and select the most informative and relevant features for accurate and efficient classification.

The state-of-the-art for leukocyte classification using machine learning can be broadly divided into two categories of works based on the type of classifier used. The first category includes classical methods of feature extraction and classification. Some notable contributions in this category are discussed as follows. In [14], a method for WBC classification was proposed which selects the color images’ eigen vectors and classifies them using a Bayesian classifier. In [15], a clustering approach was proposed that separates the cytoplasm and nucleus of leukocytes. The morphological, statistical, and geometric features were then classified using support vector machine (SVM). A mean-shift clustering based image segmentation method was proposed in [16], followed by the extraction of ensemble features of shape, texture, color, and geometry, and their classification using random forest classifier. In [17], a decision tree learning-based method was proposed for the detection of blood cancer. The proposed pipeline is based on image pre-processing and clustering steps with a random forest classifier. In [18], a segmentation approach was proposed based on finding a selective region of WBCs in the HSI color space. The nucleus and cytoplasmic granules of white blood cells were identified as colored pixels in the elliptical discriminating zone. To identify various kinds of WBCs, geometrical characteristics, color features, and LDP-based data were also retrieved and fed into several neural networks.

The second category includes the methods that use deep neural networks for feature extraction and classification. Due to the unavailability of very large datasets of WBC slides or images, the majority of the works are concentrated on using deep transfer learning. A few significant contributions are discussed here as follows. In [19], a deep learning model based on DenseNet121 CNN was proposed to classify subtypes of WBCs. The model augmentation and normalization was performed using the Kaggle dataset. In [20], dataset augmentation was performed using generative adversarial networks followed by transfer learning for WBC classification using the DenseNet169 network. The authors of [21] investigated the efficacy of pre-processing images with Gaussian and median filtering, prior to training with multiple CNN architectures such as Alexnet, ResNet50, DenseNet201, and GoogleNet20. The YOLO algorithm for WBC classification was proposed in [22].

Modern approaches utilizing transfer learning on deep CNNs are characterized by the fact that they extract a rich set of features from the images. This results in an exorbitant memory and processing power. Most often, many of these extracted deep features are redundant and do not contribute much to the classification task. Feature selection is an important step that performs dimensionality reduction of feature vectors by choosing only powerful, discriminating features. This not only reduces the processing time but also increases the accuracy of the classification task. A number of works have proposed feature selection in WBC classification, which mainly includes two types of methods, namely the filter method and the wrapper method. The filter method achieves a quick convergence to the important features; however, it does not consider the relevance between the feature subset and the classification algorithm. A wrapper approach using population-based meta-heuristics for feature selection has attracted significant research interest in recent years. This includes several evolutionary algorithms such as the genetic algorithm [23] and others [24,25,26]. A better accuracy is achieved by these methods as compared to filter-based feature selection. However, due to their iterative nature, these methods often require increased processing time. Quantum-inspired evolutionary algorithms (QIEA) [27], a class of metaheuristic algorithms inspired by the principles of quantum mechanics, provide a better exploration and exploitation of search space and hence can be an interesting solution to select powerful features while achieving a comparable or even better classification accuracy.

### Contributions

In this work, an improved WBCs classification framework was proposed, which extracts a rich set of deep features from WBC images using transfer learning on multiple deep CNNs, and performs feature selection using a customized quantum-inspired evolutionary algorithm. The contributions of this work are as follows:

1. Transfer learning was carried out utilizing two deep CNNs, namely Darknet53 and Densenet201, using a large-scale synthetic dataset of five WBC subtypes;
2. Feature vector fusion was performed to create an ensemble of extracted deep features from both networks;
3. The core contribution of this work was to model the feature selection as an optimization problem and solve it using population-based metaheuristics i.e., a quantum-inspired evolutionary algorithm (QIEA). Further, this work proposed a customized version of QIEA by performing distinct quantum rotation of individual variables of candidate solutions. The proposed algorithm effectively excludes correlated and noisy features, selecting only the most relevant features;
4. Several baseline classifiers with different kernel values were used to categorize the reduced feature set;
5. The simulation results demonstrate that the proposed method achieves a higher accuracy and a better convergence performance with a significant reduction of feature vectors as compared to several existing methods.

The remaining part of this paper is organized as follows. In Section 2, all computation steps of proposed pipeline are discussed including the dataset and customized QIEA-based feature selection. The performance results are discussed in Section 3 and, finally, Section 4 concludes the paper.

## 2. Materials and Methods

### 2.1. Dataset

In this study, a publicly available dataset of [28] was used, which has been generated synthetically from a real-world dataset [29], consisting of 6562 images representing five subtypes of WBCs, namely, monocytes, neutrophils, basophils, lymphocytes, and eosinophils.

**Figure 1:**
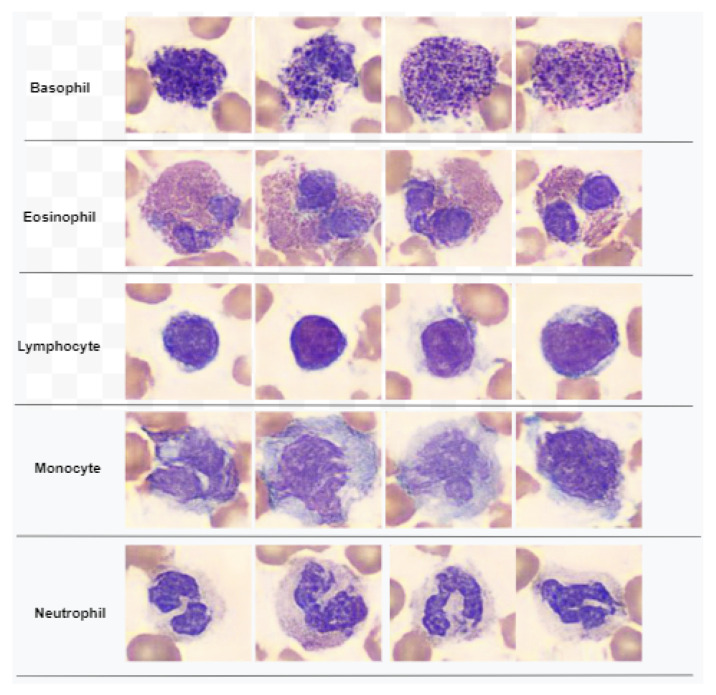
Samples of WBC images of dataset used in this work.

The original dataset of [29] was used to train the convolutional generative adversarial network (DCGAN), which then produced the dataset. The generated dataset was comprised of 5000 images with dimensions 128×128×3, with each class consisting of 1000. Few samples from all WBC classes of the dataset [28] are demonstrated in Figure 1.

### 2.2. WBC’s Classification Pipeline

**Figure 2:**
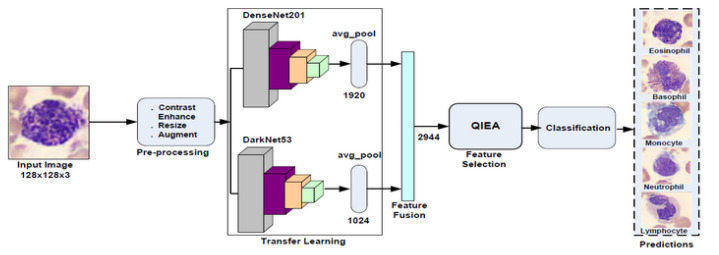
Pipeline of proposed WBCs classification system.

The main computation steps of the proposed pipeline are shown in Figure 2 and are discussed as follows.

#### 2.2.1. Preprocessing

The preprocessing step consisted of input image contrast enhancement by color histogram equalization. Normally, grayscale images are subjected to the traditional histogram equalization method, which redistributes the intensity of the images. Color histogram equalization was achieved by the conversion of RGB image space to HSV/HSI space followed by intensity enhancement, whereas hue and saturation components were preserved. The color histogram equalization was performed in the following steps.

1. Conversion of image from Red Green Blue (RGB) channels space into Hue Saturation and Intensity (HSI) space;
2. Intensity matrix calculation from HSI image;
3. Histogram equalization on the intensity matrix;
4. Replacement of HSI image intensity with histogram equalized intensity matrix;
5. HSI image conversion to RGB image.

#### 2.2.2. Feature Extraction

In this work, transfer learning for feature extraction was used with the help of two deep CNNs, i.e., DenseNet201 and DarkNet53. Both these networks are discussed as follows.

**DarkNet53** is a deep CNN originally proposed in a YOLO3 image detection framework [10]. The pretraining of this network was performed on the ImageNet [30] database. The pretrained network has the ability to classify about 1000 different image classes. Table 1 shows the layerwise details of DarkNet53 architecture. The input layer received an image of size 256×256×3. The network was composed of repeated convolution layers with filter sizes 1×1 and 3×3. The output of the convolutional layers was connected to batch normalization [31] and LeakyReLU [9] layers. Further, a residual layer was added to address the gradient disappearance problem.

In order to re-train the Darknet53 CNN, a fully connected layer (FCL) was inserted in the network in place of the “Conv53” layer. The number of outputs of FCL were kept equal to the number of WBC classes in the dataset (i.e., five classes). Further, the softmax and classification layers of the networks were also replaced by new layers. To match the network’s input layer, the dataset images were resized to 256×256×3, followed by specific augmentation operations such as random rotation and flipping. The deep feature extraction was performed from the “GlobalAvgPool” layer. A feature vector of size 1024 was obtained from Darknet53 for each image of the training as well as the testing datasets.

**DenseNet201** The dense convolutional network (DenseNet [32]) was composed of 201 layers and also trained on the Imagenet [30] dataset. The network was designed to minimize the problem of the vanishing gradient in neural networks. In DenseNet, layer concatenation was performed in such a way that each layer receives “collective knowledge” from all preceding levels. The resulting network was compact and highly efficient in terms of computational complexity and memory requirements. Table 2 shows layer details of DenseNet201.

To perform network training on our dataset, the “fc1000” layer was replaced with an FCL with five classes. A new softmax layer was also inserted along with the classification layer without class labels. In a similar fashion, the images were resized to 224×224 and augmented (rotated, flipped etc.) prior to training. The global average pooling layer was used to extract features from the trained network. A feature vector of size 1920 was obtained for each training and testing image.

#### 2.2.3. Feature Fusion

In this step, the feature vectors extracted from both networks were concatenated together to form an ensemble of features. This work adopted a serial concatenation of feature vectors. Let *X* and *Y* denote the feature vectors of size 1×m and 1×n, respectively, produced from the two deep CNNs, the fused feature vector *Z* is of dimensions 1×(m+n) and can be expressed as

(1) Z=[X,Y].

#### 2.2.4. Feature Selection Using Quantum Inspired Evolutionary Algorithm

The considerably large size of the fused feature vector extracted from above steps requires an intelligent selection of the most important features to reduce the computational complexity of the classification model while ensuring a high accuracy. As discussed earlier, in the classical filter based approach, a predefined criterion is used to select the features without consideration of the learning model. On the other hand, in the wrapper approach, the main feature selection criterion is the learning model accuracy. The main contribution of this work was to model the feature selection as an optimization problem and propose a meta-heuristic for its solution. The objective of the optimization problem was to minimize the number of selected features while maximizing the accuracy of the learning model.

**Quantum Inspired Evolutionary Algorithm**. The QIEA is a class of population-based algorithms inspired by the concepts of quantum mechanics and evolutionary computing [27]. In binary QIEA, the candidate solutions to an optimization problem, i.e., chromosomes are represented as vectors of qubits. A qubit is a vector ${\left[\alpha \;\;\beta \right]}^{T}$ such that α and β are the probabilities of observing the qubit state as |0> or |1>, respectively, complying with the condition ${\left|\alpha \right|}^{2}+{\left|\beta \right|}^{2}=1$. AqQubit state is represented as

(2) |Φ>=α|0>+β|1>.

A qubit individual (chromosome) $q_{j}$ is represented as a string of *m* Qubits as

(3) $$q_{j}=\left[\begin{array}{cccc} \alpha_{1} & \alpha_{2} & \cdots & \alpha_{m}\\ \beta_{1} & \beta_{2} & \cdots & \beta_{m}. \end{array}\right]$$

Each qubit of the chromosome represents a linear superposition of quantum states |0> and |1>. For example, consider the following three qubit system:

(4) $$q_{j}=\left[\begin{array}{ccc} \frac{1}{\sqrt{2}} & \frac{1}{\sqrt{2}} & \frac{1}{\sqrt{2}}\\ \frac{1}{\sqrt{2}} & -\frac{1}{\sqrt{2}} & \frac{1}{\sqrt{2}}. \end{array}\right]$$

Here, a linear superposition of $2^{3}=8$ states, i.e., |000>⋯|111> is represented. A greater population diversity is achieved by QIEA as compared to classical evolutionary algorithms. A quantum population Q(t) consists of *n* individual chromosomes of *m* qubits each. From the quantum population Q(t), a binary population P(t) is generated by performing the observation of the quantum state of each qubit. The evaluation phase involves the calculation of the fitness value of each individual from P(t). The recombination is performed using uniform crossover. The quantum population is then updated by applying the quantum rotation operation on each qubit as:

(5) $$\left[\begin{array}{c} \alpha_{j}^{i+1}\\ \beta_{j}^{i+1} \end{array}\right]=\left[\begin{array}{cc} Cos(\theta ) & -Sin(\theta )\\ Sin(\theta ) & Cos(\theta ) \end{array}\right]\left[\begin{array}{c} \alpha_{j}^{i}\\ \beta_{j}^{i}, \end{array}\right]$$

where θ is the rotation angle and *i* is the generation counter. The termination criterion is the maximum number of generations.

**Feature selection using QIEA**. In this study, we proposed a customized QIEA for the selection of the most powerful features from the fused feature vector. The main computation steps of the proposed QIEA are listed in Algorithm 1.

**Notations:** In Algorithm 1, double struck characters (e.g., F) are used to represent matrices and vectors, whereas normal characters denote the scalar quantities.

The inputs to the algorithm include fused feature matrix F, the label vector L, total number of generations *G*, population size *N*, and the number of variables in each solution, i.e., *M*. The matrix F has dimensions $n_{t}\times M$, where $n_{t}$ denotes the number of training images and *M* is the number of features in the fused feature vector. In Phase 1 of the algorithm, the main parameters are initialized, which include $\rho_{s}$, i.e., the probability of the best selected individuals in a population, the number of best selected features $N_{b}$, the angle of quantum rotation θ, iteration of best individual solution $X_{l}$, and global best individual $X_{gb}$. In step 4 (Phase 2), all qubits of the quantum population are initialized to equal probability $\frac{1}{\sqrt{2}}$. In steps 5–12, a binary population matrix P is generated by performing an observation procedure on the qubits.

Steps 13–39 constitute the execution phase of the algorithm. The while loop runs for *G* number of iterations (generations); during each iteration, all individuals of the population P are evaluated for their fitness value in steps 16–17. The fitness values are computed using the *CostFunction* routine, which receives as inputs the matrix F, label vector L, and binary vector A, which contains one individual of the population. Step 49 of the *CostFunction* first extracts all the features of F, whose indexes correspond to non-zero values of A, and then splits the updated feature matrix $F_{2}$ into training and testing sets. In steps 52–58 of the *CostFunction*, model training and prediction is performed using the K nearest neighbors (KNN) classifier with k=5 nearest neighbors to consider. The fitness value (cost) is computed using the classification error metric. The fitness values of all individuals returned by *CostFunction* are stored in vector Γ of the main function. In step 19 of the main function, the sort() function arranges the fitness values in ascending order along with their indexes and stores them in vectors $\Gamma_{s}$ and *I*, respectively. The individuals with minimum cost are the fittest individuals of a population. In steps 20–25, from the sorted fitness values, the iteration best and global best individuals are updated along with their fitness scores. In step 27, all probabilities of qubits are modified by applying a QIEA_Rotation function of (5).
**Algorithm 1** QIEA for feature selection.1:**Inputs:**F,L,G,N,M2:**Phase 1: Parameter Initialization**$M\leftarrow size\left(F,2\right),\;i\leftarrow 1,\;\rho_{s}\leftarrow 0.4,N_{b}\leftarrow \left\lceil \rho_{s}\times N\right\rceil \;\theta \leftarrow \frac{\pi }{4}\times rand\left(1\right),\;X_{gb}\left(1,1:M\right)\leftarrow 0,\;X_{l}\left(1,1:M\right)\leftarrow 0,\;f_{g}\leftarrow \infty ,f_{l}\leftarrow \infty$3:**Phase 2: Generate Initial Quantum Population**4:$A\left(1:N,1:M\right)\leftarrow \frac{1}{\sqrt{2}},B\left(1:N,1:M\right)\leftarrow \frac{1}{\sqrt{2}}$ 5:R←random (1:N,1:M)6:**for** x=1:N **do**7:      **for** y=1:M **do**8:           **if** R(x,y)≥B(x,y) **then**9:                P(x,y)←110:        **else**11:              P(x,y)←012:        **end if**13:    **end for**14:**end for**15:**Phase 3: Execution**16:**while** i<G **do**17:     **for** j=1:N **do**18:          A←P(j,1:M)19:         Γ(j)←CostFunction(F,L,A)20:     **end for**21:    $\left[I,\Gamma_{s}\right]\leftarrow sort\left(\Gamma \right)$22:     $P^{s}\leftarrow P\left(I\left(1:N_{b},1:M\right)\right)$23:     $X_{l}\leftarrow P^{s}\left(1,1:M\right)$24:     **if** $\Gamma_{s}\left(1\right)<f_{g}$ **then**25:          $f_{g}\leftarrow \Gamma_{s}\left(1\right)$26:          $X_{gb}\leftarrow P^{s}\left(1,1:M\right)$27:     **end if**28:     **Perform Quantum Rotation Gate Operation**29:     $B\leftarrow QIEA\_Rotation(X_{l},X_{gb},B,\theta )$30:      **Population Update**31:     R←random(1:N,1:M)32:     **for** x=1:N **do**33:           **for** y=1:M **do**34:                **if** R(x,y)≥B(x,y) **then**35:                     P(x,y)←136:                **else**37:                     P(x,y)←038:                **end if**39:           **end for**40:     **end for**41:**end while**42:**Extract Index of Best Features**43:I←1:M44:$S_{F}\leftarrow I\left(X_{gb}==1\right))$**OUTPUT: **$S_{F}$
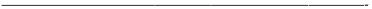
45:**Function:** CostFunction46:**Inputs:** F,L,A47:**Parameters:** $\alpha_{1}=0.99,\alpha_{2}=0.01,k=5,h_{o}=0.2$48:**if** (sum(A==1)==0) **then**49:     cost=150:**else**51:     $F_{2}\leftarrow F\left(:,\left(A==1\right)\right)$52:     $F_{train},L_{train},F_{test},L_{test}\leftarrow partition\left(F_{2},L,h_{o}\right)$53:     $A_{2}\leftarrow \left(A==1\right)$54:     $Model\leftarrow trainKNN(F_{train},L_{train},k)$55:     $L_{pred}\leftarrow predict\left(Model,F_{test}\right)$56:     $acc\leftarrow sum\left(L_{pred}==L_{test}\right)/length\left(L_{test}\right)$57:     err←1−acc58:     $f_{s}\leftarrow sum\left(A==1\right)$59:     $f_{t}\leftarrow length\left(A\right)$60:     $cost\leftarrow \alpha_{1}\times err+\alpha_{2}\times \left(\frac{f_{s}}{f_{t}}\right)$61:**end if**62:**Return:** cost
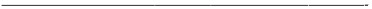
63:**Function:** QIEA_Rotation64:**Inputs:** $X_{l},X_{gb},B,\theta$65:**Initialize Parameters:**66:$a\leftarrow 0,b\leftarrow 0,a^{{}^{\prime }}\leftarrow 0,b^{{}^{\prime }}\leftarrow 0\;T\left(1:N,1:M\right)\leftarrow 0,\;B^{{}^{\prime }}\left(1:N,1:M\right)\leftarrow 0,\Phi \leftarrow 0$67:**for** x=1:N **do**68:     **for** y=1:M **do**69:           **if** ($X_{gb}\left(y\right)\&X_{l}\left(y\right)==1)$) **then**70:                Φ←θ71:           **else**72:                Φ←−θ73:           **end if**74:           b←B(x,y)75:           $a\leftarrow \sqrt{1-b^{2}}$76:           $\left[\begin{array}{c} a\prime \\ b\prime \end{array}\right]=\left[\begin{array}{cc} Cos(\Phi ) & -Sin(\Phi )\\ Sin(\Phi ) & Cos(\Phi ) \end{array}\right]\left[\begin{array}{c} a\\ b \end{array}\right]$77:           $T\left(x,y\right)\leftarrow b^{\prime }$78:      **end for**79:**end for**80:**Return:** T

The classical QIEA uses the first individual solution of the current population to compute the rotation angle. This angle is applied to all other individuals of the population. In this work, we proposed a customized version of QIEA, which uses the qubit probabilities of all individuals of the population, performing rotation on each of them with a separate rotation angle. This random rotation at the individual solution as well as at the qubit level helps the algorithm to avoid being stuck in local optima. In steps 29–39 of the main routine, a new population is generated from the updated qubit probability values. In steps 41–42, the indexes of non zero entities of global best individual $X_{gb}$ correspond to the indexes of selected features from the fused feature vector. The reduced feature vector $S_{F}$ of these selected features is returned by the algorithm.

#### 2.2.5. Classification

The reduced feature set $S_{F}$ extracted above from QIEA along with the label L are used to train multiple baseline classifiers. In this work, we used several kernels of KNN, SVM, decision tree (DT) and neural network (NN) classifiers.

## 3. Performance Results

**Figure 3:**
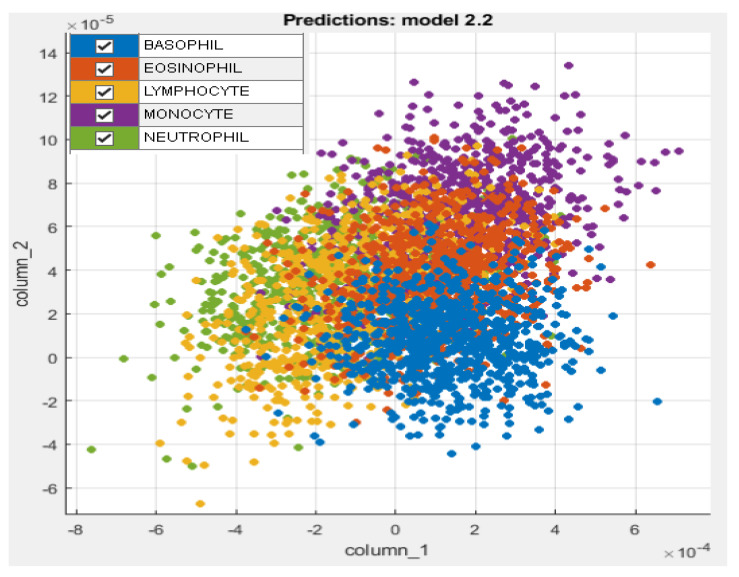
Extracted deep features from proposed QIEA feature selection method.

The proposed leukocyte classification pipeline was implemented on a Core i5 CPU with 8 GB of RAM and the Windows 10 64-bit system. The synthetic dataset of 5000 WBC images was used with a splitting ratio of 80% for training and testing, respectively. The training images were used to perform transfer learning of DenseNet201 and DarkNet53 deep models. In Table 3, important parameters of model training for deep transfer learning are listed. The training images were then applied to trained deep models, and layer activations were extracted as features. The combined feature vector of size 2944 was then subjected to the proposed QIEA feature selection method. Figure 3 demonstrates a set of reduced features extracted from the QIEA feature selection step.

**Figure 4:**
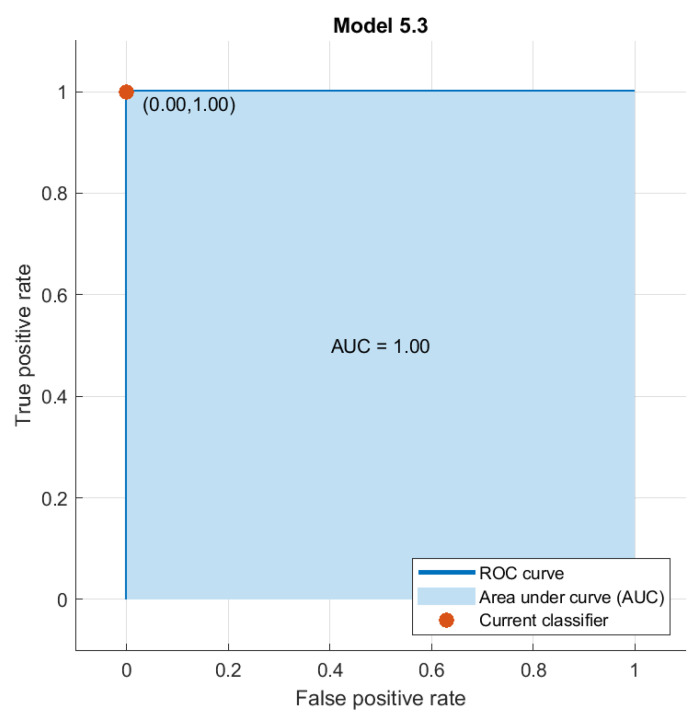
Region of convergence (test) plot of the best performing classifier, i.e., wide neural network for the proposed WBC classification pipeline.

The reduced set of selected features by QIEA was then used to train several outer classifiers such as KNN, NN, DT and SVM with multiple kernel settings. The classification results of the proposed leukocyte classification pipeline are demonstrated in Table 4 with a reduced feature vector of size 70 and various kernel settings. As evident from the Table, a maximum of 99.8% accuracy is achieved by the wide neural network classifier with 70 features extracted by QIEA out of 2944 fused features. On the other hand, the highest testing accuracy values achieved by other classifiers, i.e., SVM, KNN, and DT are 80.2%, 99.7%, and 72.4%, respectively. Figure 4 demonstrates the region of convergence (ROC) curve for the best performing classifier, i.e., the wide neural network on the reduced feature set.

Table 5 displays the testing accuracy values obtained by different classifiers using a full feature set that includes all 2944 fused features as well as reduced feature sets that were obtained through dimensionality reduction using principal component analysis (PCA) and feature selection using the proposed QIEA approach. A reduced feature vector of size 520 was obtained after PCA using a component reduction criterion of 95% explained variance. The QIEA-derived reduced feature vector outperforms the other two feature vectors, in terms of accuracy, for all classifiers examined in this work, highlighting the proposed approach’s strong feature selection capabilities.

**Figure 5:**
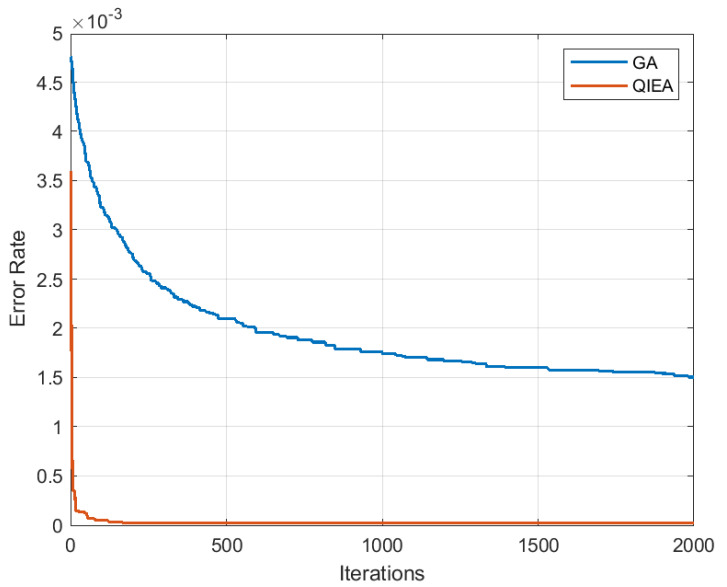
Error rate performance of proposed QIEA and genetic algorithm.

Figure 5 shows the achieved error rate of the proposed QIEA and genetic algorithm (GA) with population size N=20, and $N_{b}=10$ best selected candidates per population. The simulation for 2000 iterations shows a better error rate performance achieved by QIEA as compared to the classical GA.

**Figure 6:**
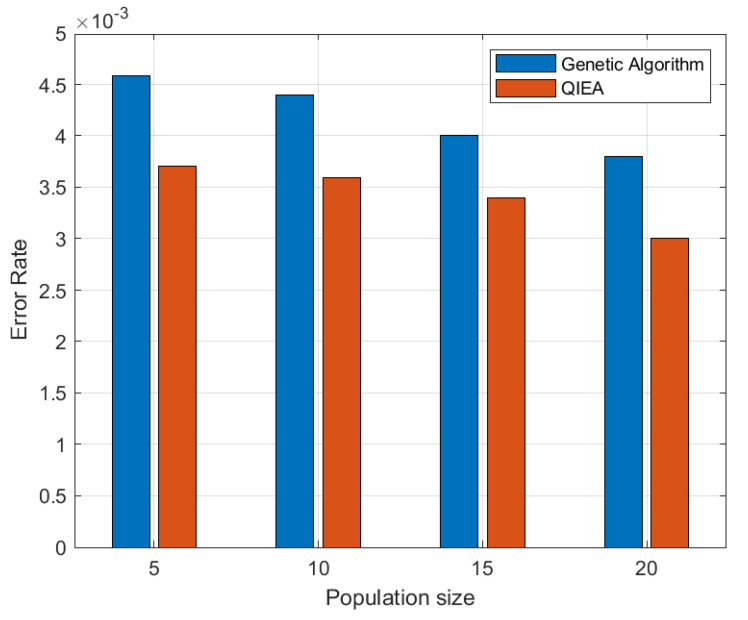
Error rate performance of QIEA and genetic algorithm with respect to population size at 20 iterations.

**Figure 7:**
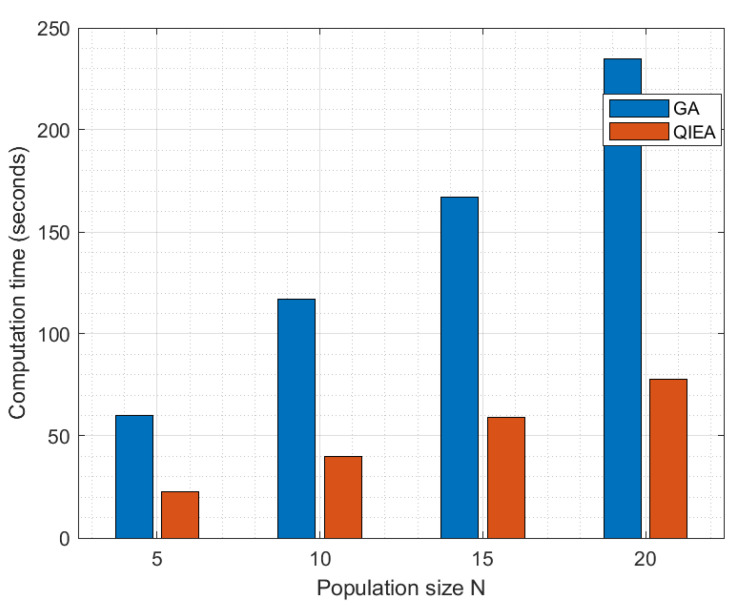
Computation time (seconds) of GA and QIEA with 200 Iterations and different population sizes.

In Figure 6, the error rate of the proposed QIEA and genetic algorithm is plotted for various values of population size *N*, with a constant value of 20 iterations. The graph first reveals that the error rate decreases with an increase in population size. Moreover, the QIEA achieves a better error rate for all values of *N*, which clearly demonstrates the effectiveness of the proposed approach. Figure 7 shows the comparison of computation time (seconds) of the classical GA and proposed QIEA for 200 iterations and different population sizes from N=5 to 20. The QIEA achieves a low computation time as compared to GA.

Table 6 presents WBC classification accuracy achieved by using a non iterative entropy-based feature selection approach and iterative feature selection using GA and QIEA methods. The entropy-based feature selection was performed using the following sequence of steps:

1. Calculate Shannon’s entropy of target variable which consists of encoded class labels of the training dataset;
2. Calculate information gain for each feature. This is carried out by calculating the conditional entropy of each feature in the feature set with respect to the target variable, using the unique values of the feature and their corresponding probabilities;
3. Subtract the calculated entropy from the target entropy to obtain the information gain for each feature;
4. Sort features by information gain in descending order;
5. Select top k features based on their information gain value;
6. Return the indices of selected k features and used these indices to extract features from the original feature set.

In this work, we selected k=1472, which corresponds to top 50% features with respect to information gain. The comparison of Table 6 clearly demonstrates that the proposed QIEA based feature selector outperforms the other two selection methods by achieving a high accuracy with a smaller feature set.

A comparison of the proposed method’s accuracy with some existing works on WBC classification that used deep learning networks and similar datasets is shown in Table 7. In comparison to prior works, the suggested technique exhibits a comparable or even superior accuracy performance with fewer features. This supports the viability of the proposed approach.

### 3.1. Discussion

In this work, we tested the WBC classification accuracy of five different feature selection approaches. In Table 5, we compared the accuracy of using a full feature set, a dimensionality reduced feature set using PCA and a QIEA-selected feature set. Similarly, in Table 6, the accuracy of iterative feature selection using GA and QIEA is compared with an entropy-based non iterative approach. A full feature set utilizes all available features for classification, potentially capturing all relevant information. However, it often includes redundant or irrelevant features which can add noise to the classification model and reduce its performance. It also causes overfitting, especially when the number of features is large as compared to the number of observations. On the other hand, PCA is an unsupervised dimensionality reduction technique which assumes that the data points have a linear structure. As shown in Figure 3, the fused features are mostly non-linearly spaced in the xy plane; therefore, PCA is poorly able to capture their relationships even with a high number of components. Evolutionary algorithms such as GA and QIEA are the supervised feature selection approaches often suited for classification tasks such as biological cell classification. A comparable accuracy is achieved by an entropy-based, non iterative feature selection. Among the approaches discussed above, QIEA achieves the best classification accuracy on the dataset used in this work, thanks to its faster convergence rate and better search space exploration capabilities.

### 3.2. Statistical Significance

In order to validate the statistical significance of our results, we used the analysis of variance (ANOVA) [37] strategy. The means of several distributions were compared in order to assess the statistical significance of our results. The classification accuracy was selected as the performance metric in the proposed framework. In order to perform ANOVA, the Shapiro–Wilk test [38] was conducted to validate the assumption of normality. The homogeneity of variance was validated using Bartlett’s test [37]. A 1% level of significance corresponding to α=0.01 was used in these tests. The means of accuracies of SVM, KNN, NNN are $\mu_{1},\mu_{2}$ and $\mu_{3}$, respectively. The null hypothesis was considered true if the Shapiro–Wilk *p*-values were less than or equal to α; the alternative hypothesis was affirmed true otherwise. The computed *p*-values of SVM, KNN, and NN classifiers were $p_{1}=0.684,p_{2}=0.723$, and $p_{3}=0.7123$, respectively. The Chi-squared probability of Bartlett’s test was $p_{ch}=0.823$. For the obtained *p*-values, we can ascertain that our accuracy values are normally distributed and have homogeneous variances.

In Table 8, the ANOVA statistical results are shown, which include the sum of squared deviation (SS), degree of freedom (df), F-statistics, mean squared error (MSE), and *p*-value. The obtained *p*-value was 0.685, which is greater than α and leads to the conclusion that the means of the three classifiers are identical.

**Figure 8:**
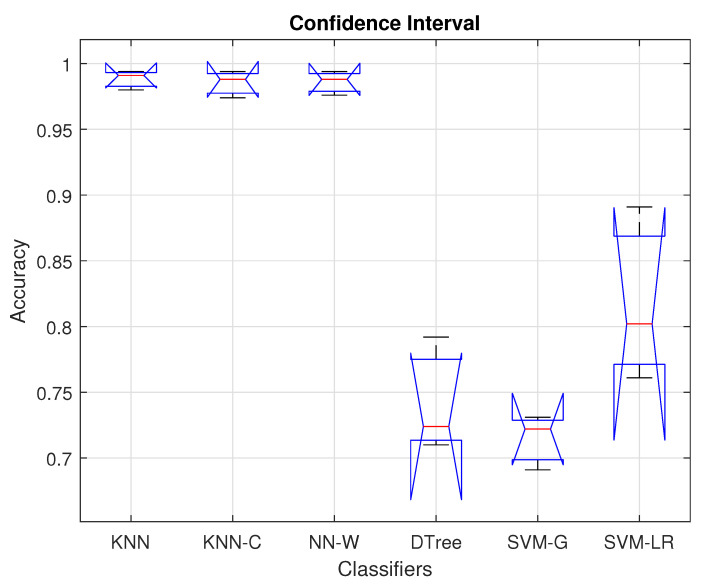
Confidence interval for selected classifiers.

In Figure 8, the confidence interval plots of accuracy values of the three selected classifiers are demonstrated. The red bars present the average accuracy, whereas the black bars present the 99% confidence limits of each classifier. Moreover, the blue bars show the lower and upper quantile points obtained by performing the above-mentioned statistical tests. The figure demonstrates that the KNN and wide-NN classifiers achieve a higher average accuracy with a relatively smaller confidence interval size as compared to the other classifiers. The quantile points of each classifier lie within their respective confidence limits. The higher *p*-values resulting from these quantile points lead to the acceptance of null hypotheses, which means significant differences in the accuracy distribution of the classifiers.

## 4. Conclusions

The classification of WBCs is crucial in the diagnosis of certain blood disorders, particularly leukemia. In modern approaches for leukemia detection using transfer learning on deep neural networks, feature extraction is a crucial task to reduce the ‘curse of dimensionality’ while achieving a high accuracy. This work proposed an improved WBCs classification pipeline in which deep transfer learning was first applied as a feature extractor followed by an efficient quantum-inspired feature selection algorithm. The proposed customized version of a quantum-inspired evolutionary algorithm avoids the local optima and achieved an accuracy of 99.8% with a reduction of more than 95% in the size of the feature vector. The error rate performance of the proposed algorithm demonstrates its effectiveness as compared to classical population based meta-heuristics for feature selection. The proposed WBC classification pipeline can be integrated as a sub-system of a clinical grade setup such as automated image flow cytometry.

## Figures and Tables

**Table 1:** Layerwise details of DarkNet53.

Layer Type	Filters	Filter Size	Stride Size	Repeat	Output Size
Input	-	-	-	-	224×256
Convolutional	32	3×3	1	1	256×256
Convolutional	64	3×3	2	1	128×128
Convolutional	32	1×1	1	1	
Convolutional	64	3×3	1		
Residual					128×128
Convolutional	128	3×3	2	1	64×64
Convolutional	64	1×1	1	2	
Convolutional	128	3×3	1		
Residual					64×64
Convolutional	256	3×3	2	1	32×32
Convolutional	128	1×1	1	8	
Convolutional	256	3×3	1		
Residual					32×32
Convolutional	512	3×3	2	1	16×16
Convolutional	256	1×1	1	8	
Convolutional	512	3×3	1		
Residual					16×16
Convolutional	1024	3×3	2	1	8×8
Convolutional	512	1×1	1	4	
Convolutional	1024	3×3	1		
Residual					8×8
GlobalAvgPool					
Fully Connected	1000				
Softmax					

**Table 2:** Layerwise details of DenseNet201 network.

Type of Layer	Composition	Repeat Factor	Output Size
Input	–	–	224×224
Convolution	Conv (7×7), stride 2		112×112
MaxPool	(3×3), stride 2		56×56
Dense Block 1	Conv (1×1)	6	
	Conv (3×3)		56×56
Transition Layer 1	Conv (1×1)	1	56×56
	Avg Pool (2×2), Stride 2		28×28
Dense Block 2	Conv (1×1)	12	
	Conv (3×3)		28×28
Transition Layer 2	Conv (1×1)	1	28×28
	Avg Pool (2×2), Stride 2		14×14
Dense Block 3	Conv (1×1)	48	
	Conv (3×3)		14×14
Transition Layer 3	Conv (1×1)	1	14×14
	Avg Pool (2×2), Stride 2		7×7
Dense Block 4	Conv (1×1)	32	
	Conv (3×3)		7×7
Classification Layer	7×7 Global Avg. Pool		
	1000D fully Connected, softmax		1×1

**Table 3:** Main model training parameters for DenseNet201 and DarkNet53 transfer learning.

Parameter	Value	Parameter	Value
Kernel	sdgm	Learning Rate	1× $10^{-4}$
Execution Environment	Auto	MiniBatch Size	20
MaxEpochs	5	Validation Frequency	30
Dropout rate	0.1	Stride Size	1

**Table 4:** Classification test results of proposed WBCs classification system with different classifiers.

Classifier	Feature Vector Size	Accuracy %	Precision	Recal	F1 Score	Sensitivity
KNN Coarse	70	99.7	0.997	0.997	0.996	0.994
KNN Cosine		99.1	0.99	0.997	0.997	0.991
NN Wide		99.8	0.997	0.998	0.998	0.995
Decision Tree (Medium)		72.4	0.73	0.726	0.725	0.742
SVM (Gaussian)		72.2	0.882	0.72	0.741	0.734
SVM (Regression)		80.2	0.890	0.80	0.842	0.887

**Table 5:** Accuracy comparison of WBC classification using full feature set, reduced feature set using PCA based dimensionality reduction, and reduced feature set using QIEA-based feature selection.

Classifier	Dimensionality		Feature Selection		Using	
	Reduction		Using		Full Feature	
	Using PCA		QIEA		Set	
	Feature Vector Size	Accuracy %	Feature Vector Size	Accuracy %	Feature Vector Size	Accuracy %
KNN Coarse		97.1		99.7		98
KNN Cosine		97		99.1		98.1
NN Wide	520	99.2	70	99.8	2944	99.1
Decision Tree (Medium)		71		72.4		70.1
SVM (Gaussian)		70.8		72.2		60.1
SVM (Regression)		81		80.2		79

**Table 6:** Comparison of the accuracy of three feature selection methods used in WBC classification pipelines: non-iterative entropy-based feature selection and wrapper feature selection using GA and QIEA.

Classifier	Feature Selection		Feature Selection		Entropy Based	
	Using		Using		Feature	
	GA		QIEA		Selection	
	Feature Vector Size	Accuracy %	Feature Vector Size	Accuracy %	Feature Vector Size	Accuracy %
KNN Coarse	289	98	70	99.7	1472	94.3
KNN Cosine		96.9		99.1		92.5
NN Wide		98.6		99.8		92.8
Decision Tree (Medium)		72		72.4		70
SVM (Gaussian)		71.4		72.2		71.1
SVM (Regression)		78		80.2		78.2

**Table 7:** Performance comparison of proposed method with some existing works. ×: Not done, N.A: Information not available.

Work	Deep Learning Model	Feature Selection	Feature Vector Size	Classifier	Accuracy %
[33]	GoogleNet, ResNet-50	Maximal Information Coefficient, Ridge Regression Model	755	Quadratic Discriminant Analysis	97.95
[34]	AlexNet	×	1000	CNN	98.4
[35]	PatternNet fused ensemble of CNNs	×	N.A	CNN	99.90
[36]	ResNet and Inception	Hierarchical Approach	N.A	ResNet and Inception	99.84
**This Work**	**DenseNet201 and DartkNet53**	**QIEA**	**76**	**SVM, KNN, NN, DT**	**99.8**

**Table 8:** Statistical test results based on ANOVA using accuracy metric.

V-Source	SS	df	MSE	F-Statistics	*p*-Value
Between	6.815 $\times \;10^{-5}$	2	2.6258 $\times \;10^{-5}$	0.34	0.685
Within	6.2136 $\times \;10^{-4}$	6	8.8614 $\times \;10^{-5}$	-	-
Total	6.1259 $\times \;10^{-4}$	8	-	-	-

## Data Availability

The data for this work shall be available upon request.

## Abbreviations

The following abbreviations are used in this manuscript:

WBC	White blood cell
CNN	Convolutional neural network
DNN	Deep neural network
SVMs	Support vector machines
QIEA	Quantum Inspired Evolutionary Algorithm
NN	Neural Network
DT	Decision Tree
KNN	K-nearest neighbors
TPR	True positive rate
FNR	False negative rate

## References

[1] Farag M.R., Alagawany M. (2018). Erythrocytes as a biological model for screening of xenobiotics toxicity. Chem. Biol. Interact..

[2] Rezatofighi S.H., Soltanian-Zadeh H. (2011). Automatic recognition of five types of white blood cells in peripheral blood. Comput. Med. Imaging Graph..

[3] Weatherspoon D. What to Know about White Blood Cells. https://www.medicalnewstoday.com/articles/327446#types-and-function.

[4] Mathur A., Tripathi A.S., Kuse M. (2013). Scalable system for classification of white blood cells from Leishman stained blood stain images. J. Pathol. Inform..

[5] LeCun Y., Bengio Y., Hinton G. (2015). Deep learning. Nature.

[6] Bengio Y., Courville A., Vincent P. (2013). Representation learning: A review and new perspectives. IEEE Trans. Pattern Anal. Mach. Intell..

[7] Nguyen L.D., Lin D., Lin Z., Cao J. Deep CNNs for microscopic image classification by exploiting transfer learning and feature concatenation. Proceedings of the 2018 IEEE International Symposium on Circuits and Systems (ISCAS).

[8] Simonyan K., Zisserman A. (2014). Very deep convolutional networks for large-scale image recognition. arXiv.

[9] He K., Zhang X., Ren S., Sun J. Deep residual learning for image recognition. Proceedings of the IEEE Conference on Computer Vision and Pattern Recognition.

[10] Redmon J., Farhadi A. (2018). Yolov3: An incremental improvement. arXiv.

[11] Howard A.G., Zhu M., Chen B., Kalenichenko D., Wang W., Weyand T., Andreetto M., Adam H. (2017). Mobilenets: Efficient convolutional neural networks for mobile vision applications. arXiv.

[12] Chollet F. Xception: Deep learning with depthwise separable convolutions. Proceedings of the IEEE Conference on Computer Vision and Pattern Reco Gnition.

[13] Shin H.C., Roth H.R., Gao M., Lu L., Xu Z., Nogues I., Yao J., Mollura D., Summers R.M. (2016). Deep convolutional neural networks for computer-aided detection: CNN architectures, dataset characteristics and transfer learning. IEEE Trans. Med. Imaging.

[14] Sanei S., Lee T.K. T: Cell recognition based on pca and bayesian classification. Proceedings of the 4th International Symposium, ICA 2003.

[15] Sarrafzadeh O., Rabbani H., Talebi A., Banaem H.U. Selection of the best features for leukocytes classification in blood smear microscopic images. Proceedings of the Medical Imaging 2014: Digital Pathology, SPIE.

[16] Ko B., Gim J., Nam J. (2011). Cell image classification based on ensemble features and random forest. Electron. Lett..

[17] Kumar P. (2021). Matlab Based Potent Algorithm for Wbc Cancer Detection and Classification. Biomed. Pharmacol. J..

[18] Su M.C., Cheng C.Y., Wang P.C. (2014). A neural-network-based approach to white blood cell classification. Sci. World J..

[19] Sharma S., Gupta S., Gupta D., Juneja S., Gupta P., Dhiman G., Kautish S. (2022). Deep learning model for the automatic classification of white blood cells. Comput. Intell. Neurosci..

[20] Almezhghwi K., Serte S. (2020). Improved classification of white blood cells with the generative adversarial network and deep convolutional neural network. Comput. Intell. Neurosci..

[21] Yildirim M., Çinar A. (2019). Classification of White Blood Cells by Deep Learning Methods for Diagnosing Disease. Rev. D’Intell. Artif..

[22] Alam M.M., Islam M.T. (2019). Machine learning approach of automatic identification and counting of blood cells. Healthc. Technol. Lett..

[23] Gupta D., Agrawal U., Arora J., Khanna A. (2020). Bat-inspired algorithm for feature selection and white blood cell classification. Nature-Inspired Computation and Swarm Intelligence.

[24] Liu J., Lin Y., Li Y., Weng W., Wu S. (2018). Online multi-label streaming feature selection based on neighborhood rough set. Pattern Recognit..

[25] Ahmad R., Awais M., Kausar N., Akram T. (2023). White Blood Cells Classification Using Entropy-Controlled Deep Features Optimization. Diagnostics.

[26] Shahzad A., Raza M., Shah J.H., Sharif M., Nayak R.S. (2022). Categorizing white blood cells by utilizing deep features of proposed 4B-AdditionNet-based CNN network with ant colony optimization. Complex Intell. Syst..

[27] Han K.H., Kim J.H. (2002). Quantum-inspired evolutionary algorithm for a class of combinatorial optimization. IEEE Trans. Evol. Comput..

[28] Jung C., Abuhamad M., Mohaisen D., Han K., Nyang D. (2022). WBC image classification and generative models based on convolutional neural network. BMC Med. Imaging.

[29] (2019). The Catholic University of Korea Institutional Review Board. https://bit.ly/2YrlQPl.

[30] ImageNet. http://www.image-net.org.

[31] Ioffe S., Szegedy C. Batch normalization: Accelerating deep network training by reducing internal covariate shift. Proceedings of the International Conference on Machine Learning. PMLR.

[32] Huang G., Liu Z., Van Der Maaten L., Weinberger K.Q. Densely connected convolutional networks. Proceedings of the IEEE Conference on Computer Vision and Pattern Recognition.

[33] Toğaçar M., Ergen B., Cömert Z. (2020). Classification of white blood cells using deep features obtained from Convolutional Neural Network models based on the combination of feature selection methods. Appl. Soft Comput..

[34] Hegde R.B., Prasad K., Hebbar H., Singh B.M.K. (2019). Feature extraction using traditional image processing and convolutional neural network methods to classify white blood cells: A study. Australas. Phys. Eng. Sci. Med..

[35] Wang J.L., Li A.Y., Huang M., Ibrahim A.K., Zhuang H., Ali A.M. Classification of white blood cells with patternnet-fused ensemble of convolutional neural networks (pecnn). Proceedings of the 2018 IEEE International Symposium on Signal Processing and Information Technology (ISSPIT).

[36] Habibzadeh M., Jannesari M., Rezaei Z., Baharvand H., Totonchi M. Automatic white blood cell classification using pre-trained deep learning models: Resnet and inception. Proceedings of the Tenth International Conference on Machine Vision (ICMV 2017).

[37] Akram T., Laurent B., Naqvi S.R., Alex M.M., Muhammad N. (2018). A deep heterogeneous feature fusion approach for automatic land-use classification. Inf. Sci..

[38] Akram T., Naqvi S.R., Haider S.A., Kamran M., Qamar A. (2020). A novel framework for approximation of magneto-resistance curves of a superconducting film using GMDH-type neural networks. Superlattices Microstruct..

